# The individual, the government and the global community: sharing responsibility for health post-2015 in Vanuatu, a small island developing state

**DOI:** 10.1186/s12939-015-0244-1

**Published:** 2015-10-24

**Authors:** Claire Ibell, Simon A. Sheridan, Peter S. Hill, John Tasserei, Marie-France Maleb, Jean-Jacques Rory

**Affiliations:** School of Public Health, Public Health Building, University of Queensland, Herston Road, Herston, QLD 4006 Australia; Health Promotion Unit, Public Health Department, Private Mail Bag 9009, Port Vila, Efate Vanuatu; Sanma Provincial Health Office, PO Box 265, Luganville, Espiritu Santo Vanuatu

**Keywords:** Sustainable development goals, Post-2015 health agenda, Pacific Island Countries, Small island developing states

## Abstract

**Introduction:**

The end of 2015 will see the creation of the sustainable development goals – the new global framework for development. The process of creating universally relevant goals has involved community consultation throughout the world. Within this process it is vital that Pacific Island countries are included as they face particular development challenges due to their size and geographical location. As small island developing states, many Pacific Island countries struggle to overcome high rates of poverty and poor health outcomes. In order to include Pacific voices in the new health related sustainable development goals, Vanuatu was selected as a representative of the Pacific for this qualitative study. This paper presents the perspectives of communities throughout Vanuatu on their essential health needs and how best to meet them.

**Methods:**

This paper examines the perspectives of 102 individuals from throughout Vanuatu. Ten focus group discussions and 2 individual interviews were conducted within communities in September 2013. Discussions focused on community perceptions of health, essential health needs, and responsibility in achieving health needs. Discussions were audio recorded and transcribed. The transcripts were then analysed using a theoretical thematic approach in order to identify central themes and subthemes.

**Results:**

Individuals in this study demonstrated a comprehensive understanding of health, defining health in a holistic manner. Participants identified clear environmental and societal factors that impact upon health, and emphasized failures within the current health system as important barriers to attaining good health. Participants described the challenges faced in taking responsibility for one’s health, and pointed to both the government and the international community as key players in meeting the essential health needs of communities.

**Conclusions:**

As a small island developing state, Vanuatu faces accentuated development challenges – particularly as globalisation and climate change progress. The individuals and communities in this study demonstrate a clear understanding of their needs, and show a strong desire for change. They point to both the government and the international community to assist in meeting health needs, and stress that respect for traditional governance and community involvement in decision-making are vital in this process. In order to ensure that the new health goals effectively meet local needs in Vanuatu such factors must be incorporated into policy and implementation decision-making.

## Introduction

This year represents great change in the global development agenda. The end of 2015 will see a transition from the Millennium Development Goal (MDG) framework to a new and revitalised vision. The Sustainable Development Goals (SDGs) provide the framework for global development over the next 15 years. The SDGs aim to be inclusive, transformative and global; appropriate for developed and developing countries alike [1]. Importantly, the goals have been created through an inclusive and participatory process, aiming to ensure the voices of individuals and communities at all levels of society are considered [1, 2].

Within the new development agenda it is vital that voices from the Pacific are heard. Pacific Island countries (PICs) face particular development challenges including small populations, geographical isolation and vulnerability to climate change [3]. A number of PICs are classed as ‘least developed countries’ by the United Nations (UN) [4], and one quarter of Pacific Islanders live below national basic needs poverty lines [3]. Economic development within these countries is often limited due to poor institutional capacity and isolation from international markets [5, 6]. Further, many PICs face a double burden of disease [7]. Communicable diseases remain prevalent in a number of PICs, however there is also a growing non-communicable disease (NCD) ‘crisis’ [8], which will not only increase strain on under-resourced health systems, but also affect the economic prospects of many countries in the region. It is thus imperative that the special development challenges of PICs are included in the post-2015 development debate in order to ensure their particular needs are addressed.

The present qualitative study was completed for the Goals and Governance for Health project (Go4Health). Go4Health is a global consortium of academics and public health specialists working to inform the debate on the post-2015 health related development goals [9]. In particular, Go4Health is committed to ensuring that the health goals are developed in collaboration with the communities that they will most effect [10]. In order to achieve this, Go4Health has conducted consultations with a number of communities around the world. Consultations aim to engage local community members, particularly those of vulnerable and marginalised populations, in discussions around their essential health needs. In order to include the viewpoints of a Pacific Island nation, Vanuatu was selected for community consultation in the region. This paper presents the results of ten focus group discussions and two individual interviews carried out within communities throughout Vanuatu in 2013. The objectives of this paper are to:

- present the essential health needs and expectations of local communities within Vanuatu in order to contribute to post-2015 planning, and
- understand the challenges faced in Vanuatu at both local and national levels in regards to meeting health needs and creating a clear and effective development agenda.

The present work builds on that of Sheridan and colleagues (2014), who analysed the health related views of a cohort of youth that made up part of this broader qualitative study [11]. The adolescent perspectives are included in this analysis as they necessarily are a part of a whole community cross section which make up this qualitative study. However, for an in depth analysis of the youth perspectives the reader is referred to the previous work of Sheridan and colleagues (2014).

## Background

Vanuatu is a country of around 276,000 people [12], situated in the South-western Pacific. The country is made up of 83 islands, the majority of which are inhabited [13]. Around 75 % of Vanuatu’s population live in rural or remote areas [13, 14], which can result in difficult and costly service provision. Rural to urban migration is on the rise, however this is not without its own development issues [14, 15]. Vanuatu falls into the World Bank’s lower middle income category, and has a GDP per capita of approximately $3100 USD [16]. Agriculture, fishing and tourism are important industries within the country [17], and according to the 2009 National Census, around 40 % of the country’s labour force work in subsistence agriculture [13].

Vanuatu, like many of the PICs, has made mixed progress in achieving the MDGs [18]. In terms of health, the country’s under-five mortality rate is markedly decreasing [19–21], however the prevalence of underweight children is increasing [19, 20]. Maternal deaths remain higher than the country’s target of less than 3 per year, with an estimated 6 in 2009 [18]. Further, the contraceptive prevalence rate remains low (38 % in 2013) [21], and the country has a high population growth rate of 2.3 % per annum [21].

Vanuatu is currently experiencing an ‘epidemiological transition’ and faces a challenging double burden of disease – with both communicable diseases and NCDs prominent [21, 22]. Particular communicable diseases of concern include tuberculosis (TB), malaria and sexually transmitted infections (STI) [21]. However, despite severe resource constraints, the Government of Vanuatu is committed to improving the health status of the population and has made consistent progress in reducing the communicable disease burden. For example, the prevalence of TB within the country is lower than the regional average [23], and the incidence of malaria has reduced over tenfold since 1990 [20]. However, although the country has a low incidence of HIV, like many PICs risk factors for it’s spread remain high [18]. Poor use of condoms, low levels of correct knowledge around HIV, and a weak health system could contribute to an increase in HIV incidence in the future [21]. Further, sexually transmitted infections are highly prevalent in Vanuatu - in fact the country has some of the highest rates in the Pacific [24].

Despite the health issues posed by communicable diseases, it is the non-communicable disease burden that is the most pressing in Vanuatu. The country is in the midst of the ‘NCD crisis’ reported by many PICs - as global economies impact on Vanuatu and the dependence on cheap, imported foodstuffs has increased, so too have the risk factors for NCDs [25–27]. According to the WHO STEPwise Approach to Surveillance of Risk Factors for NCDs survey (STEPS) published in 2013, NCDs are responsible for around 70 % of deaths in Vanuatu [28]. In order to address this burden the country has included NCDs as a priority in the Priorities and Action Agenda of 2006 – 2015 [27]. Further, through Pacific Health Ministers Meetings Vanuatu, along with other PICs, is committed to taking inter-sectorial approaches to curb the incidence of chronic disease and bring the crisis in the Pacific to international attention [8].

In addition to meeting domestic needs such as improving the health status of the population, the Government of Vanuatu recognises the importance of contributing to international decision making in relation to both Pacific issues and those of global importance. However, as a small island developing state the country faces particular development challenges. With extremely limited human and financial resources, high vulnerability to economic and environmental shocks, a small domestic market and a dependence on foreign aid [6, 29], achieving both domestic needs and international obligations poses a considerable challenge indeed. In order to assist in addressing such challenges Vanuatu is part of the Small Island Developing States (SIDS) network [6]. The network provides a platform for SIDS countries throughout the world to collaborate and problem-solve around shared development challenges, and to work together to bring these challenges to global attention.

The UN has also recognised the special challenges faced by SIDS nations, and included Vanuatu in their ‘Consultations on Localising the Post-2015 Agenda’. The UN consultations were carried out in order to ensure local community input into the development of the SDGs from various regions around the world [30]. The consultations were conducted in 2014 in the country’s capital, Port Vila, and included representatives from local authorities, civil society organisations, traditional leaders, non-government organisations (NGOs) and development partners [31]. Although the Vanuatu dialogues contribute important viewpoints on development challenges and strategies to overcome these, the consultations were held in an urban centre which may have precluded some communities from participating. The present study sought to engage local communities from urban, rural and remote locations and targeted health discussion in particular, thus contributing an inclusive, health related perspective to the post-2015 discussions. The UN Vanuatu consultations will be further addressed in the discussion section of this paper.

## Methods

Data collection for the present qualitative study was conducted within Vanuatu in September of 2013. Ten focus group discussions and 2 individual interviews were carried out in communities throughout Vanuatu by the research team, involving a collaboration of the University of Queensland and the Vanuatu Ministry of Health. Communities were selected from urban, rural and remote areas in order to reflect the vast geographical spread of the population. Three islands were visited in order to conduct focus group discussions – Efate (centrally located), Espiritu Santo (north), and Tanna (south). Communities on each island were selected both on the basis of geographical location and on permission granted by chiefs and community leaders.

A total of 102 participants were included - ten focus group discussions and two individual interviews. Individuals of various ages were included, with two focus groups conducted solely with adolescent participants. Both male and female community members participated, and where possible group discussions were followed by smaller, separate male and female discussions.

Methods of recruiting participants differed between communities. Two focus groups were conducted with young people selected from two secondary schools. The secondary schools were approached and the study was explained to the school principals. The principals were asked to select 10 students who demonstrated positive attitudes (whether evidenced by leadership qualities, or ability or enthusiasm in any particular area). This was to ensure students were not selected on the basis of academic ability alone. There is an inherent bias, however, toward more educated youth simply because the participants were secondary school students. A further two focus groups were conducted with parents of primary school children in different communities. The principals of two schools were approached and the study objectives explained. The principals were asked to request parents of primary school children to represent their local communities in the focus groups. A further six focus groups were conducted with adult community members from various villages across the three islands visited. The research team contacted either village chiefs or provincial councillors in each community in order to explain the study and invite communities to take part. Community members were then selected on the basis of availability in order to participate in the focus group discussions. Finally, two individual interviews were conducted which allowed inclusion of the perspectives of a female public health worker and a female community member in the study. Local public health staff shared responsibility for ensuring that sampling was culturally sensitive, ensuring broad coverage but respecting local cultural norms.

Focus groups were carried out in English and Bislama using translators where necessary. Table 1 (appendix) presents the characteristics and location of each focus group and individual interview, along with language used.

**Table 1:** Summary of focus group discussions and individual interviews conducted throughout Vanuatu

Focus group no.	Location, name of island	Number of participants			Participant type	Language
		Total	Female	Male		
1	Urban, Efate	*n* = 10	*n* = 5	*n* = 5	Secondary school students	English
2	Rural, Efate	*n* = 12	*n* = 6	*n* = 6	Parents of primary school children	Bislama
3	Rural, Efate	*n* = 10	*n* = 4	*n* = 6	Adult community members	Bislama
4	Remote, Tanna	*n* = 10	*n* = 5	*n* = 5	Adult villagers	Bislama
5	Remote, Tanna	*n* = 10	*n* = 5	*n* = 5	Adult villagers	Bislama
6	Rural, Tanna	*n* = 8	*n* = 1	*n* = 7	Adult community members	English, Bislama
7	Remote, Espiritu Santo	*n* = 11	*n* = 5	*n* = 6	Adult villagers	Bislama
8	Rural, Espiritu Santo	*n* = 11	*n* = 6	*n* = 5	Adult community members	Bislama
9	Urban, Espiritu Santo	*n* = 8	*n* = 6	*n* = 2	Parents of primary school children	Bislama
10	Urban, Espiritu Santo	*n* = 10	*n* = 5	*n* = 5	Secondary school students	English
11	Urban, Espiritu Santo	*n* = 1	*n* = 1		Female community member	Bislama
12	Urban, Efate	*n* = 1	*n* = 1		Female public health worker	English
Total		*n* = 102	*n* = 50	*n* = 52		

Modified from: Sheridan S, Brolan C, Fitzgerald L, Tasserei J, Maleb M, Rory J, Hill P. Facilitating health and wellbeing is “everybody’s role”: youth perspectives from Vanuatu on health and the post-2015 sustainable development goal agenda. International Journal for Equity in Health. 2014;13;80

A question guide developed by the Go4Health project was used to guide focus group discussions and individual interviews. Questions focused on the areas of 1) community understandings of health, 2) determinants of health, 3) essential health needs and their provision, 4) roles and responsibilities of relevant actors, and 5) community participation in decision-making [32].

The discussions were audio-recorded and then transcribed from the recordings. The transcripts were then analysed using a theoretical thematic analysis approach [33]. Theoretical thematic analysis is an approach used to identify themes within data that is guided by the researcher’s specific interest or research question. That is, data was analysed with a specific research question in mind, rather than the research question developing during the process of data analysis [33]. Analysis was conducted through reading the transcripts a number of times, then manually coding data into groups of interesting ideas and quotes. These groups were then analysed to identify commonalities and differences, and resulting patterns were grouped together into major themes and sub-themes which are presented in this paper.

## Ethical considerations

Ethical approval was granted through the University of Queensland School of Public Health’s Human Research Ethics Committee, and by the director of Public Health within Vanuatu’s Ministry of Health. Further, participants were provided with information regarding the reasons for carrying out the study, assurance of confidentiality, and their right to withdraw at any point. Written informed consent was obtained from participants who were literate, and verbal consent recorded on a digital recorder was obtained from those who were not.

## Results

Four major themes were drawn from the analysis of focus group and individual discussions, and are presented below. The themes illustrate the way in which participants viewed health, along with clear environmental and societal factors identified as impacting upon health. Participants emphasized failures within the health system to respond to the essential needs of communities as important barriers in attaining good health, and described the challenges in taking responsibility for health outcomes.

### Definitions of health – health as a holistic concept

Although some individuals – particularly male youth - expressed a largely physical interpretation of health, the vast majority viewed health in a holistic manner. Health was seen to incorporate physical, mental, spiritual and social aspects, with participants from both urban and rural areas explicitly describing each component:

*“Mentally I have to think properly, my mind must be at a good stage. Physically I must do some exercise, for instance walking, gardening, and not sit down too much. Spiritually I must go to church, family worship and take part in church activities. Socially – do not be alone at your house but go and be friends with your neighbour. Make relationships with your friends because if you stay alone then you are not healthy. You cannot say that if you are not sick then you are healthy, no, it is the four aspects of life – spiritual, mental, physical and social. If you have these four aspects of life then you can say you are healthy.” (Adult female participant from urban area on Espiritu Santo)*

*“When I was a kid I did not understand the meaning of health. My understanding of health was brushing my teeth and eating good food. But when I grew up then I realized that what I was doing was not health, because I was just doing part of it….health is spiritual, mental, social and physical. Only then can you say you are healthy.” (Adult male participant from rural community on Efate)*

Both youth and adult participants identified having a positive attitude, being confident in oneself, and having a purpose in life as contributing factors to good health. However, one notable difference between youth and adult perspectives was the view by many adults that traditional values and being part of a community are integral to health. One female participant explains:

*“I come from an area where it is a community, it’s a big community, people living together, you have different communities from different islands living together so, I think what are the factors that make people continue to be healthy or living together, first probably it’s about how family gets together….It’s about how one cares for another – we do things together – if there are issues and somebody has a problem communities and families support them.” (Female participant working in health in an urban area of Efate)*

Adult participants from at least one discussion group on every island placed importance on Pacific values such as welcoming behaviour, smiling, appropriate dress, and traditional food preparation. One participant discussed how cultural practices should be incorporated into one’s idea of health:

*“Pacific education must be emphasized, especially with our cultural ethics. That’s very healthy because a lot of cultural practices like smiling and being kind, inviting people home, being friendly, treating people well, it’s all good practices.” (Adult female participant from a rural area on Tanna)*

Further, every focus group emphasized the importance of a functioning health system in maintaining health. However, the importance of traditional values was also evident in the use of traditional medicine in combination with western medicine:

*“Plenty of women are sick, some become paralysed but they have come to receive medical, natural and spiritual prayer and plenty have recovered…Some women in the community come across some sickness like stroke. They have used health medication, natural medicine and prayer.” (Adult female participant in a remote community of Espiritu Santo)*

*“All the time when a Ni-Vanuatu is sick he does not go to a health facility straight away, he goes to either his traditional healer or to his pastor, or his church elder.” (Female participant working in health in an urban area of Efate)*

The ‘traditional’ consisted of a combination of island beliefs, such as ‘kastom’ medicine and Chieftain governance structures, and Christian values - a remnant of years of colonisation by both the British and French. There was a strong recognition of the importance of village chiefs among youth participants, however in contrast to adult groups Christian values were not explicitly highlighted by youth.

In addition to this holistic concept of health, participants showed a comprehensive understanding of factors that contribute to physical health. For example exercise, sufficient rest and a good diet were all highlighted as important in keeping one’s body healthy. In particular, health was strongly associated with a sense of personal and environmental cleanliness:

*“The environment must be clean for us to breathe fresh air so we can stay healthy. Clean your surroundings, inside and outside the house.” (Adult female participant from a rural community on Espiritu Santo)*

*“For a person that is not healthy means his home is not clean, environment is not clean and his surroundings are not clean – this person’s life will be infected very easily with sickness.” (Adult male participant from remote community on Tanna)*

Finally, evidence of the effects of increasing globalisation could be seen at the local level as participants identified the negative impact of imported food within communities. The importance of a balanced diet was highlighted in 11 out of the 12 discussions, and a move back to local island foods was linked strongly to health. Participants in three focus groups (across rural and urban areas) specifically spoke of an increased incidence of NCDs within their communities due to the consumption of imported foods, and emphasis was consistently placed on the need to eat locally grown products in order to maintain health:

*“More local food is good for the health of the kids and the community as a whole. Today we feed our kids with more imported food but we have to discourage this and encourage parents to provide local food.” (Adult male participant from a rural community on Espiritu Santo)*

*“Good health is local food.” (Adult male participant from rural community on Efate)*

Individuals in three focus groups pointed out that it can be challenging to encourage the consumption of local foods due to both a lack of education, and the cheap cost of imported foods when compared to local products.

### How the environment impacts upon health – water and sanitation challenges

Based on their concepts of health and the factors that contribute to good health, participants were asked about the essential health needs of their communities. Improved water and sanitation systems were consistently identified as one of the greatest needs in every focus group. As an island nation, water is integral to the way of life in Vanuatu. Participants in half of all focus groups made mention of the importance of the sea to support livelihoods, to swim in to remain healthy and to be used for washing when fresh water is not available. Moreover, fresh water was discussed in all focus groups, regardless of locality, as a scarce resource. One participant expressed concern at the growing population and thus increased pressure on already limited water supplies:

*“Water is one of the most important needs. We have one source but we need more water tanks as at the moment we only have one tank. The community is also facing a shortage of water. As the population is growing, we need more water tanks. At the moment three houses share one tap which is not healthy. One tank is feeding the whole population of Fanafo.” (Adult male participant from a remote community on Espiritu Santo)*

Two participants described a complete lack of water within their communities at times. As the population of Vanuatu continues to grow rapidly and the effects of climate change progress, this situation may become more frequent. The following participants describe their experiences:

*“There’s no access to water on that island, so you have to get water up from the mountain on the mainland to where I’m from. There’s a total of 500 people in our village, and they’re only small but desperate for water from the mountain. It’s like that in small places. It comes to the village and gets divided into five taps. Five taps will cater for 500 people. Sometimes the water doesn’t run that strong so there’s no water for that community…Sometimes when there’s no water, we have to swim in the sea and get the water there…we don’t drink water, we only drink coconut. Yes, that’s our main source if there is no water.” (Adolescent male participant in focus group conducted in urban Efate)*

*“During the dry season there is no more water, because they have to fetch water from a creek or something like that. It is very distant for washing, drinking and cooking and sometimes they will have no bath, no water to drink. So they should have a water tank to accommodate the community when there is no water.” (Adult female participant from urban area on Espiritu Santo)*

In addition to a shortage of water, water quality was also identified as a problem in many communities. Participants in nearly half of the focus groups, covering all three islands, requested assistance from either the government or health workers in order to improve water quality. Further, at least one participant from each island linked poor water systems with inadequate sanitation and hygiene. ‘Cleanliness’ was not only seen as a defining factor of health, but also translated into an essential health need within communities through the need for improved sanitation systems:

*“One factor that contributes to poor health is poor toilets, this community uses local toilets (bush toilets)…Bush toilets are toilets that don’t use water because we do not have a proper water system.” (Adult male participant from a rural community on Efate)*

*“The advice to stay healthy is to keep our bodies clean, and to be clean we need clean water, but we do not have clean water nor enough water.” (Adult female participant from remote community on Espiritu Santo)*

Appropriate sanitation systems were not only seen as improved toilet systems, but included factors such as personal hygiene and waste management. At least one participant from every group described the importance of hygiene in maintaining health; from sleeping in clean, well-ventilated areas, to hand hygiene and appropriate food preparation. Further, individuals from all focus groups on the island of Efate described the need for improved waste management in communities. Although an issue in both rural and urban areas, one young participant emphasized the poor state of waste management and infrastructure in informal urban settlements. She described a lack of proper housing and rubbish disposal, resulting in the dumping of rubbish in waterways and along roadsides. A number of participants linked these issues of poor sanitation and waste disposal with specific illnesses such as malaria, TB and diarrhoea. Moreover, individuals expressed a clear understanding of the need to maintain a clean home and community in order to prevent insect breeding sites and the spread of malaria. Further, diarrhoea was identified as an ongoing problem amongst both children and adults, exacerbated by poor sanitation and a weak health system.

### How the health system impacts upon health - inadequate health system capacity

Poor health system capacity was consistently identified as an enormous barrier to achieving good health. Particular areas of focus included inadequate health facilities, poor transport systems, a shortage of trained health workers, and a lack of health awareness programs in communities. There appeared to be a tension between individuals feeling personal or community responsibility in achieving their health needs, and a frustration with the lack of government services to allow them to do so. This discord can be illustrated through the strong emphasis placed on the need for increased health education – this issue was highlighted in three quarters of discussion groups spanning urban, rural and remote areas. Participants were asking for the knowledge to be able to improve their own health - health awareness programs were seen as a means of empowering and educating communities. One young participant explains:

*“Awareness that the people from the health services provide for the people in the islands is another factor that contributes to keep the community healthy. It educates the people. Even if they do not talk about everything but they touch on some special areas…it will help them to improve their health, and keep themselves and their communities healthy.” (Adolescent female participant from focus group conducted in urban Efate)*

One woman expresses her frustration at the infrequent nature of health campaigns:

*“Health awareness has to be carried out maybe every six months as it is being a long time before health workers come back to do another awareness session.” (Adult, rural community on Efate)*

Participants spoke of the need for education in a range of areas - from basic hygiene and food preparation, to taking care of infants. Some individuals mentioned the need for awareness programs on specific diseases such as HIV, cervical cancer and sexual and reproductive health. One group of women in an urban setting explained that domestic violence is common, in terms of both physical and emotional violence. They described a lack of control over accessing health services, sexual matters and contraception, and household matters such as finances. The women specifically pointed to increased awareness campaigns within the community, for both women and men, as a means of changing this violence.

In addition to health education, the need to improve health system infrastructure was identified as essential. The need for accessible health facilities for the rural population was highlighted in three quarters of the discussion groups, including at least one group from each island. One woman emphasized the need for a facility in her community, particularly to improve maternal and child health:

*“This community has some members with various sicknesses, and when women are pregnant some deliver their babies just in this village because we do not have a health centre. This community is having access only to the Vila Central Hospital. We urgently need a health centre in this community. Also other sicknesses like kids infected with sores go untreated because we have to travel to the Vila Central Hospital, and some community members do not have the finances to travel to and from the hospital.” (Adult participant, rural Efate)*

The challenges faced due to a lack of health facilities in rural areas are compounded by poor transport systems. A lack of vehicles, the cost of transport and poor roads and travel routes all contribute to the problem. At least one participant from each island described instances of patients dying due to an inability to reach a health facility. Others pointed out that they acknowledge medical advice to get regular check ups, however they are unable due to a lack of finances or an inability to reach a health centre. Two adult participants from Tanna describe this challenge between taking personal responsibility for one’s health, and the constraints faced in doing so due to a lack of services:

*“We don’t have ‘prevention is better than cure’. It is almost too late for us every time so we just need the cure. Every time that we end up at the hospital it’s too late for us. That always happens in the Pacific Islands. For any disease, every disease – high sugar, diabetes, cancer. Most people end up in hospital with the doctor telling them ‘you have cancer’. It’s very late. They should have been admitted before. It’s not the policy here to have medical check ups.” (Adult male participant in rural community on Tanna)*

*“I guess that is linked to not enough medical facilities, when you can have check ups on the spot. And the transport to the health centre, they don’t want to worry about that until later. Later when the time comes, it’s too late - he or she has to go to the hospital. It could be the ambulance to the hospital. And that is far too late for the check up.” (Adult male participant in rural community on Tanna)*

Participants from every island further identified a lack of health workers as an important barrier to attaining good health. Individuals in a quarter of focus groups described long waiting times when accessing services, sometimes having to return home without treatment. Further, at least one participant from every island pointed out that there is a particular shortage of doctors, leaving nurses overburdened with extremely large caseloads. It was acknowledged, however, that health workers are trying to do the best they can in an environment with limited resources. Both adult participants and younger individuals suggested that the government must create greater incentives for health workers to move to rural areas, and that health staff must be supported in their roles:

*“What I would like to suggest is maybe they can increase salaries in rural areas so that doctors may want to go back and work there. Nowadays no one wants to work in the rural areas.” (Adolescent male participant in urban Efate)*

*“The government has to take responsibility to ensure that health workers are happy and comfortable in their working environment.” (Adult female participant in urban area of Espiritu Santo)*

### Responsibility for health – the individual, the government and the international community

When identifying where responsibility lies in order to meet health needs, the majority of groups recognised the place of personal agency and collective community responsibility for health, however all pointed to the essential responsibility of the government to provide the necessary infrastructure. The following participants explain this dynamic:

*“The first one [responsible for health] is the government. Financially it’s the government… for health infrastructure. Then number two is individually. Everyone is responsible for their own health.” (Adult male participant from rural community on Tanna)*

*“[When initiating health related projects] Local authorities must go to the national Government for financial assistance, but the community will assist in manpower. The same goes for health facilities – but the financial assistance must come from the Government.” (Adult female participant from urban area of Espiritu Santo)*

In over three quarters of the discussions there was a sense of the importance of collaboration and shared responsibility for the collective health of the community. This was the case in urban, rural and remote settings. One female participant states:

*“Decision-making has to be agreed upon by all the organisations in the community. For example the chiefs, church leaders, youth leaders, women leaders and others in order to make the health needs come through….The community lives with the health issues and the villagers must raise their concerns with the chiefs in order to figure out how we can stop the issues.” (Adult participant, rural Efate)*

However, it was consistently recognised that communities cannot meet the health needs of their people without support from the government. Ultimately, participants from every focus group felt that responsibility lies with the Government of Vanuatu. A number of individuals expressed frustration at the lack of basic services in many communities and the apparent lack of concern of the authorities, as described by the following two participants:

*“I think this is one big thing that the government just seems to be avoiding. It’s doing everything in the world, signing every agreement and convention in the world – I think we are one of the countries that have signed 92 conventions and we can only remember three. We’d like to do everything, to keep up with the Joneses and the Smiths, but it’s not getting us anywhere. The essentials, the government doesn’t care about.” (Adult female participant from rural community on Tanna)*

*“The Department of Health has not been doing enough to come to the community level to monitor what health needs the communities are facing.” (Adult male participant from rural Efate)*

Despite this perspective, some individuals pointed out that Vanuatu simply doesn’t have the finances nor the human resources to ensure essential health needs are met. The importance of aid was mentioned in three rural and remote villages. Further, two young participants showed remarkable insight into the development challenges faced by the government. While acknowledging the importance of taking responsibility for health and wellbeing they explain that they feel it is unrealistic to expect Vanuatu to be able to meet basic health needs without financial and technical assistance from other countries.

Finally, the traditional Chieftain governance structure of the country was seen as vital in ensuring the health needs of communities are met. Both rural and urban groups, youth and adult participants alike placed value on the role of chiefs in decision-making and guidance, and as links between communities and the government.

## Discussion

### Summary of main findings

A number of key findings can be highlighted from this study. Firstly, participants demonstrated a comprehensive understanding of health, with various societal factors contributing to good health. Not only did individuals show a clear understanding of physical factors that contribute to health such as a balanced diet, exercise and hygiene, but health was also seen to incorporate tradition and culture. This could be seen in a number of areas including the importance of local island food, the presence of traditional medicine in the midst of a ‘western’ health system, and the sustained reliance on traditional governance. Further, the importance of community was highlighted in the Ni-Vanuatu interpretation of health. Not only was socialising and being supported by one’s community identified as a component of being healthy, but the majority of focus groups also described a collective community responsibility for health.

Secondly, individuals in every focus group consistently highlighted a lack of basic services as one of the greatest barriers to health. Widespread lack of consistent access to clean water, poor sanitation systems, and inadequate health services arose as major limitations in achieving health. Interestingly this did not differ between islands nor locations – urban, rural and remote communities all described similar challenges. This may in part be due to the fact that many people who live in urban areas have moved from rural or remote villages, thus participants in urban groups may not provide a true representation of uniquely urban issues. Further, people living in informal urban settlements often do not have access to the basic infrastructure in place in formal urban settings [34].

A third key finding is the recognition of the importance of collaboration and shared responsibility for health, alongside the need for the government to provide the necessary infrastructure. The vast majority of focus group discussions involved an interplay between individuals recognising their own responsibility in ensuring the good health of themselves and their communities, and the challenges in achieving this with little government support in place. In order to meet health needs, many groups identified the need for collaboration between stakeholders and respect and utilisation of traditional governance structures and community leaders.

Overall there were no stark differences in issues raised between islands or localities. The only significant difference between issues raised by men and women related to gender specific sexual and reproductive health issues. Women in five focus groups discussed uterine cancer, urinary incontinence post childbirth or other reproductive health issues as common health problems for women. Further, one group of women in an urban area raised the issue of domestic violence, pointing to the need for increased education campaigns targeted at both men and women to address such issues. Finally, one notable difference between adult versus youth focus groups was the emphasis that a number of adults placed on Christian values in relation to health. In contrast, youth did not make mention of such values, however they did emphasize respect for chiefs and the importance of community.

### Discussion of key issues

As highlighted in every focus group across all islands and localities, the state of basic service provision throughout Vanuatu is poor. Data from the most recent DHS reinforce participant perspectives. According to the survey although 90 % of the population have ‘access to an improved water source’, only around 30 % of rural households have access to a piped water source [21]. Further, issues remain around water quality, with only sporadic and uncoordinated water quality testing being carried out [35]. In terms of sanitation, the DHS reports that approximately 50 % of households within the country have appropriate sanitation facilities [21]. This poor level of coverage contributes to the ongoing incidence of diarrhoea and waterborne illnesses. As climate change, population increases and urbanisation continue to progress, so too will the challenges in achieving appropriate water and sanitation services for all. The Government of Vanuatu has made some effort to improve this situation. In order to improve the management of water resources, a National Water Strategy for the period 2008-2018 was created with the assistance of development partners. The strategy was designed to create an inclusive, participatory approach and provides a framework for an integrated water management system for the country [35]. However, according to a national assessment carried out in 2013, implementation of the strategy has been limited due to capacity constraints [29].

As participants in this study further emphasized, access to health services remains limited, particularly in rural areas. Currently the health system is made up of a hierarchical system of services. Aid posts and dispensaries exist at the most local level of the population and feed into health clinics and larger referral hospitals in main centres [36]. There is also a focus on preventative care and addressing the social determinants of health through the Healthy Islands Policy [37]. However, although the structure of the health system is built on a sound base, the efficacy of the system remains limited by resource constraints, poor human resource capacity, and geographical barriers [21, 31, 38]. There is a severe shortage of health professionals, particularly in rural areas, and poor referral systems between differing levels of health facilities [34].

The Vanuatu Ministry of Health has created a Health Sector Strategy for the period of 2010-2016 with the broad goals of providing equitable, quality services and improving the health status of the population. As part of this strategy the government aims to achieve a decentralised health system with the planning, management and provision of services controlled at the provincial level [36]. A number of participants in the present study reinforced the need for this to happen. However, the country is yet to achieve a quality, decentralised system and sustained commitment and resources are required if progress is to be made.

In order to meet these development challenges participants in this study emphasized the need for community involvement in decision-making, and stressed the vital importance of traditional Chieftain governance structures. The importance of including stakeholders, and in particular chiefs, in decision-making and policy planning is also explicitly stated in the country’s Priorities and Action Agenda [27], thus the government does recognise the importance of such involvement. Further, the country faces particular challenges in providing the basic services described above. Although the government must fulfil their responsibilities, one must also consider the country’s status as a small island developing state, and as a ‘least developed country’. The government has a responsibility to provide the population with basic health services and infrastructure, however they must do this from an extremely limited financial and human resource base. The wide geographical distribution of the population results in high service delivery costs [29]. A small population and resource base, along with isolation from international markets and high transport costs further inhibit the country from developing a strong economy [6]. Such factors result in amplified development challenges that the country cannot overcome alone. Moreover, in addition to meeting domestic needs, it is essential that Vanuatu maintain an international voice in order to contribute to decision-making around vital issues affecting the Pacific – perhaps most notably climate change. However, maintaining international obligations puts increased strain on an already under-resourced system. Some participants in this study showed insight into the development challenges faced by their government, and pointed to the international community as essential in assisting the country to move forward. This call reinforces that made by Vanuatu and other SIDS nations through the SAMOA pathway (SIDS Accelerated Modalities of Action pathway) of 2014. The SAMOA pathway was adopted by the UN in November 2014, with the purpose of “reaffirming commitment to the sustainable development of small island developing States” [5]. Within this document, SIDS nations have called for ongoing international assistance in order to make progress on sustainable development, stating “we recognize the ownership and leadership of small island developing states in overcoming some of these challenges [to sustainable development], but stress that in the absence of international cooperation, success will remain difficult” [5]. This call by no means diminishes the responsibility of the Government of Vanuatu to lead with sound and just governance, however it does emphasize that collaboration is required. If the post-2015 development agenda is to be truly equitable, the international community must play a vital role in assisting SIDS nations to progress toward sustainable development.

### How do these findings contribute to current knowledge?

As described earlier in this paper, the UN included Vanuatu in their ‘Consultations on Localising the Post-2015 Agenda’, carried out in 2014. The consultations created discussion broadly around the MDGs and lessons learned, the proposed SDGs, and the role of key stakeholders in implementation of the post-2015 agenda [31]. The discussions were not health specific but rather discussed the future development agenda as a whole. Further, consultations were targeted at a policy and legislation level, thus perhaps making them somewhat inaccessible to individuals within local communities. However, many of the challenges identified in implementing an effective development agenda are in line with issues described by participants in this study. For example, poor infrastructure and basic service delivery, a lack of information management and poor information sharing, poor governance and political instability, and a lack of capacity at national, provincial and local levels were all identified in the UN consultations as obstacles to effective development. Participants in the present study similarly emphasized a lack of basic services, poor governance and inadequate capacity as limitations to achieving good health. However, in contrast to the UN consultations, the present study sought to understand the beliefs of individuals and communities around health, thus providing an insight into daily challenges faced and possible areas of focus for future health policy and interventions. For example, health was understood as multi-faceted and complex, with various social, spiritual, mental and physical components. Further, the presence of traditional medicine in the midst of a ‘western’ health system was evident, and although a sound health system was seen as vital, it was also acknowledged that ‘kastom’ medicine and local healers have a role to play in the health of communities. There is a need to recognise that for health policy and interventions to be effective they must be culturally sensitive and appropriate.

The UN consultations suggested a number of factors in moving forward that correlate with the findings of the present study. It was emphasized that the Vanuatu government must continue to commit to the current plan of decentralisation of governance, and local and provincial authorities must be up-skilled to make this process effective. Further, strong links with traditional governance were identified as essential, including increased support and development training for village chiefs. Community participation in decision-making and inclusion in information sharing was also noted as an important focus. Finally, donor coordination and alignment with local and national priorities was seen as key to the implementation of an effective development agenda [31]. Similar factors were endorsed by participants in the present study, particularly around decentralisation of governance, respect for chiefs and local community leaders, and community involvement in decision-making.

### Study limitations

While communities selected for this study were chosen in order to accurately reflect the vast geographical spread of the population of Vanuatu, individual participants were selected on the basis of availability and permission granted by community leaders. As participants within communities were not selected strictly randomly this may introduce some bias into the results. The study does however include a relatively large number of participants and attempts to capture perspectives from a range of urban, rural and remote communities. Further, when considering the perspectives of other Pacific Island countries and SIDS nations, it must be kept in mind that this study explores the views of communities only within Vanuatu. The results are thus not generalizable to other populations. However, although the results are not generalizable across other PICS and SIDS nations, they do raise some valid points that can be considered in the development agenda of such countries.

### Policy implications of the research

The individuals in this research show that the people of Vanuatu have a clear perspective of their needs, and a strong desire to share in affecting change. Participants are requesting both government and international assistance in allowing them to meet their health needs. They are calling for integration of traditional governance structures with the structures of a democratic state, and a changing relationship with international donors. In order to ensure that the new health goals meet the needs of such communities we must listen to their call. As the new health goals are agreed upon, the UN focus on consultation regarding the development agenda must be sustained with a focus on implementation, and continue to include local stakeholders at every level. In order to ensure that the voices of local communities in Vanuatu are incorporated into health policy the following recommendations are made:

- the Government of Vanuatu must continue to work towards a decentralised health system with value placed on community input into decision-making
- traditional governance must continue to be integrated into the country’s governance system, with greater utilisation of chiefs and community leaders in assisting in the meeting of health needs
- health promotion and policy must respect and incorporate local understandings of health, for example the importance of community social structures

## Conclusion

Vanuatu is a small island developing state facing many development challenges. As the impacts of globalisation increase, the population faces new and varied challenges in meeting their health needs. The individuals and communities in this study demonstrate a clear understanding of their needs, and show a strong desire for change. They point to both the government and the international community to assist in meeting health needs, and stress that respect for traditional governance and community involvement in decision-making are vital in this process. In order to ensure that the new health goals effectively meet local needs in Vanuatu such factors must be incorporated into policy and implementation decision-making.

## Abbreviations

MDG: Millennium development goals
SDG: Sustainable development goals
PIC: Pacific Island Country
UN: United Nations
NCD: Non-communicable disease
GDP: Gross domestic product
DHS: Demographic and health survey
TB: Tuberculosis
STI: Sexually transmitted infection
WHO: World Health Organisation
HIV: Human immunodeficiency virus
STEPS: STEPwise approach to surveillance of risk factors for NCDs survey
SIDS: Small island developing state
NGO: Non-government organisation
Ni-Vanuatu: Local person from Vanuatu
SAMOA: SIDS accelerated modalities of action pathway
